# Supplementary material for: The interplay between ranking and communities in networks
Source: Sci Rep. 2022 May 30;12:8992. doi: 10.1038/s41598-022-12730-3 (PMC9151911; doi:10.1038/s41598-022-12730-3)
Supplement: Supplementary file 1 — Supplementary Information. [file 41598_2022_12730_MOESM1_ESM.pdf]

Supplement: "The interplay between ranking and communities in networks"

Laura Iacovissi[1, 2, +] **and Caterina De Bacco**[1, *]

[1]Max Planck Institute for Intelligent Systems, Cyber Valley, Tübingen 72076, Germany
[2]Bosch Industry on Campus Lab, University of Tübingen
[+]laura.iacovissi@uni-tuebingen.de
[*]caterina.debacco@tuebingen.mpg.de

S1 Controlling sparsity

In order to control the average degree value given by the ranking and community structures, we can introduce two parameters c_{SR}, c_{MT} s.t. $c_{SR} = c$ and $w_{kh} = c_{MT}\hat{w}_{kh}$,$\forall k,h$. We assume that the average degree is composed by three contributes, i.e.:

$$\langle k\rangle N = \langle k_{SR}\rangle N + \langle k_{MT}\rangle N + \varepsilon N = \mathbb{E}\left[\sum_{ij} A_{ij}\right] , \tag{1}$$

with ε noise term given by the outgroup interactions. Under the XOR model, we have:

$$\mathbb{E}\left[\sum_{ij} A_{ij}\right] = \sum_{ij}\sum_{\sigma} P(\delta_{\sigma_i\sigma_j})\mathbb{E}_{A|\sigma}[A_{ij}] = 2\mu(1-\mu)\delta_0 N^2 + \left(\mu^2+(1-\mu)^2\right)\sum_{ij}\mu S_{ij} + (1-\mu)M_{ij} \tag{2}$$

Hence, imposing the equivalence for each term:

$$\varepsilon = 2\mu(1-\mu)\delta_0 N , \tag{3}$$

$$c_{SR} = \frac{\langle k_{SR}\rangle N}{\mu\left(\mu^2+(1-\mu)^2\right)\sum_{ij} e^{-\beta H_{ij}}} , \tag{4}$$

$$c_{MT} = \frac{\langle k_{MT}\rangle N}{(1-\mu)\left(\mu^2+(1-\mu)^2\right)\sum_{ijkh} u_{ik} v_{jh}\hat{w}_{kh}} . \tag{5}$$

Notice that this formulation assumes that the outgroup interaction parameter δ_0 has to respect the following bound:

$$\delta_0 \leq \frac{\langle k\rangle}{2\mu(1-\mu)N} . \tag{6}$$

S2 Mapping to an Ising model

In order to define this mapping, it is convenient to rewrite the posterior distribution of σ such that:

$$P(\sigma|A,\theta) \propto \exp(\log P(\sigma,A|\theta)) =: \exp(-H_A(\sigma|J,h))) \tag{7}$$

for some energy function

$$H_A(\sigma|J,h) = -\sum_i h_i(\theta)\, s_i(\sigma_i) - \sum_{ij} J_{ij}(\theta)\, s_i(\sigma_i)\, s_j(\sigma_j) , \quad 2\sigma_i - 1 = s_i(\sigma_i) \in \{-1,1\} . \tag{8}$$

We recall that:

$$\log P(\sigma, A|\theta) = \sum_i \log\left(\mu^{\sigma_i}(1-\mu)^{1-\sigma_i}\right) \sum_{ij} \log\left[\text{Pois}(A_{ij}; S_{ij})^{\sigma_i \delta_{\sigma_i\sigma_j}} \text{Pois}(A_{ij}; M_{ij})^{(1-\sigma_i)\delta_{\sigma_i\sigma_j}} \text{Pois}(A_{ij}; \delta_0)^{1-\delta_{\sigma_i\sigma_j}}\right] . \quad (9)$$

From now on, we will use $\bar{S}_{ij}, \bar{M}_{ij}, \bar{\lambda}_0$ to denote the logarithm of the poisson distributions in A_{ij} with means $S_{ij}, M_{ij}, \lambda_0$. In addition, the dependence on θ and σ willl be avoided, in order to make the notation easier to read.

Consider the first summation appearing in Eq. (9). It is equal to:

$$\sum_i \sigma_i \log\mu + (1-\sigma_i)\log(1-\mu) = \sum_i \frac{s_i+1}{2}\log\mu + \left(1 - \frac{s_i+1}{2}\right)\log(1-\mu) \quad (10)$$

$$= \sum_i \frac{1}{2}\left(\log\mu - \log(1-\mu)\right)s_i + \text{const} , \quad (11)$$

where the constant is not relevant since it will be discarded when normalizing the Boltzmann distribution. Hence we have

$$h_i^{(1)} \equiv \frac{1}{2}\left(\log\mu - \log(1-\mu)\right) \quad (12)$$

as the first component of the h_i field. To obtain the J_{ij} and $h_i^{(2)} = h_i - h_i^{(1)}$ fields, we consider the second summation of Eq. (9), which corresponds to $\log P(A|\sigma,\theta)$:

$$\log P(A|\sigma,\theta) = \sum_{ij} \sigma_i \delta_{\sigma_i\sigma_j}\bar{S}_{ij} + (1-\sigma_i)\delta_{\sigma_i\sigma_j}\bar{M}_{ij} + (1-\delta_{\sigma_i\sigma_j})\bar{\lambda}_0 \quad (13)$$

$$= \sum_{ij}\left(\frac{s_i+1}{2}\right)\left(\frac{s_i s_j+1}{2}\right)\bar{S}_{ij} + \left(1-\frac{s_i+1}{2}\right)\left(\frac{s_i s_j+1}{2}\right)\bar{M}_{ij} \quad (14)$$

$$+ \left(1 - \frac{s_i s_j+1}{2}\right)\bar{\lambda}_0 \quad (15)$$

$$= \sum_{ij} s_i s_j \frac{\bar{S}_{ij} + \bar{M}_{ij} - 2\bar{\lambda}_0}{4} + \sum_i s_i \sum_j \frac{\bar{S}_{ij} + \bar{S}_{ji} - \bar{M}_{ij} - \bar{M}_{ji}}{4} \quad (16)$$

$$+ \frac{\lambda_0}{2}N^2 + \sum_{ij}\frac{\bar{S}_{ij} + \bar{M}_{ij}}{4} \quad (17)$$

Now we have:

$$J_{ij} = \frac{\bar{S}_{ij} + \bar{M}_{ij} - 2\bar{\lambda}_0}{4} \quad (18)$$

$$h_i^{(2)} = \frac{1}{4}\sum_j(\bar{S}_{ij} + \bar{S}_{ji} - \bar{M}_{ij} - \bar{M}_{ji}) \quad (19)$$

The final Hamiltonian is:

$$H_A(s|J,h) = \sum_{i,j} J_{ij} s_i s_j + \sum_i (h_i^{(1)} + h_i^{(2)}) s_i \quad (20)$$

where the J_{ij} are asymmetric.

S3 Mean-field approximation for q

We decribe here the procedure followed for computing the approximation of the variational distribution q under mean-field assumption. Firstly, we assume that we can factorize it as $q(\sigma) = \prod_i q_i(\sigma_i)$, $q_i(\sigma_i) = \text{Be}(Q_i)$. Hence, the collection of means $\{Q_i\}_i$ fully charecterises the distribution q. We notice that we can write our objective function as

$$\mathscr{L}(q,\theta) = Q_i\, \mathbb{E}_{j\neq i}\big[\log P(A,\sigma_i=1,\sigma_{j\neq i}|\theta)\big] + (1-Q_i)\, \mathbb{E}_{j\neq i}\big[\log P(A,\sigma_i=0,\sigma_{j\neq i}|\theta)\big] + H_b(Q_i) + \text{const}\,. \tag{21}$$

Maximizing w.r.t. Q_i gives the equation:

$$\log\left(\frac{Q_i}{1-Q_i}\right) = \mathbb{E}_{j\neq i}\big[\log P(A,\sigma_i=1,\sigma_{j\neq i}|\theta)\big] - \mathbb{E}_{j\neq i}\big[\log P(A,\sigma_i=0,\sigma_{j\neq i}|\theta)\big]\,, \tag{22}$$

which is solved for

$$Q_i = \frac{\exp\left(\mathbb{E}_{j\neq i}\big[\log P(A,\sigma_i=1,\sigma_{j\neq i}|\theta)\big] - \mathbb{E}_{j\neq i}\big[\log P(A,\sigma_i=0,\sigma_{j\neq i}|\theta)\big]\right)}{1+\exp\left(\mathbb{E}_{j\neq i}\big[\log P(A,\sigma_i=1,\sigma_{j\neq i}|\theta)\big] - \mathbb{E}_{j\neq i}\big[\log P(A,\sigma_i=0,\sigma_{j\neq i}|\theta)\big]\right)} \tag{23}$$

$$= \frac{\exp\left(\mathbb{E}_{j\neq i}\big[\log P(A,\sigma_i=1,\sigma_{j\neq i}|\theta)\big]\right)}{\exp\left(\mathbb{E}_{j\neq i}\big[\log P(A,\sigma_i=1,\sigma_{j\neq i}|\theta)\big] + \mathbb{E}_{j\neq i}\big[\log P(A,\sigma_i=0,\sigma_{j\neq i}|\theta)\right)} \tag{24}$$

$$= \frac{\exp\left(\mathbb{E}_{j\neq i}\big[\log P(\sigma_i=1|A,\theta,\sigma_{j\neq i})\big]\right)}{\exp\left(\mathbb{E}_{j\neq i}\big[\log P(\sigma_i=1,|A,\theta,\sigma_{j\neq i})\big] + \mathbb{E}_{j\neq i}\big[\log P(\sigma_i=0|A,\theta,\sigma_{j\neq i})\right)}\,. \tag{25}$$

Since the formula for $P(A,\sigma|\theta)$ is known, we can compute this quantity and obtain $Q_i = q_i(\sigma_i = 1)$. Using the $\delta_{\sigma_l\sigma_k}$ parametrization, neglecting self-loops and discarding the terms not dependent on σ_i, we finally obtain the following formula:

$$Q_i = \frac{f_{i1}}{f_{i1}+f_{i2}}\,, \tag{26}$$

$$f_{i1} = \mu \prod_{j\neq i} [\mathrm{Pois}(A_{ij};S_{ij})\mathrm{Pois}(A_{ji};S_{ji})]^{Q_j}\, [\mathrm{Pois}(A_{ij};\lambda_0)\mathrm{Pois}(A_{ji};\lambda_0)]^{(1-2Q_j)}\,, \tag{27}$$

$$f_{i2} = (1-\mu)\prod_{j\neq i} [\mathrm{Pois}(A_{ij};M_{ij})\mathrm{Pois}(A_{ji};M_{ji})]^{Q_j-1}\,. \tag{28}$$

S4 $\mathscr{L}$ maximization under mean-field assumption

Here we report the computations for defining the EM algorithm updates. The goal is to maximise the following quantity:

$$\mathscr{L}(q,\theta) = \sum_\sigma q(\sigma)\, \log\frac{P(\sigma,\theta|A)}{q(\sigma)} = \sum_\sigma q(\sigma)\, \log P(A|\sigma,\theta) + \sum_\sigma q(\sigma)\, \log P(\sigma|\mu) + H(q) \tag{29}$$

where H is the Entropy function. As a first step, we can write the log-likelihood term more explicitly as

$$\sum_\sigma q(\sigma)\, \log P(A|\sigma,\theta) = \sum_{i,j,\sigma}\Big[\sigma_i q(\sigma)\, \delta_{\sigma_i\sigma_j} \log \mathrm{Pois}(A_{ij};S_{ij}) + (1-\sigma_i)\, q(\sigma)\, \delta_{\sigma_i\sigma_j} \log \mathrm{Pois}(A_{ij};M_{ij}) + q(\sigma)(1-\delta_{\sigma_i\sigma_j})\, \log \mathrm{Pois}(A_{ij};\delta_0)\Big]\,. \tag{30}$$

Now consider the first term in Eq. (30).

$$\sum_{i,j,\sigma} \sigma_i q(\sigma)\, \delta_{\sigma_i\sigma_j} \log \mathrm{Pois}(A_{ij};S_{ij}) = \sum_{ij} Y_{ij} \log \mathrm{Pois}(A_{ij};S_{ij})\,; \tag{31}$$

$$Y_{ij} := \sum_\sigma \sigma_i q(\sigma)\, \delta_{\sigma_i\sigma_j}\,. \tag{32}$$

Since we work under the MF assumption, the following equality holds:

$$Y_{ij} = \sum_\sigma \sigma_i q(\sigma)\, \delta_{\sigma_i\sigma_j} = \sum_\sigma q(\sigma)\left(2\sigma_i^2\sigma_j - \sigma_i^2 - \sigma_i\sigma_j + \sigma_i\right) = \mathbb{E}[\sigma_i]\,\mathbb{E}[\sigma_j] = Q_iQ_j\,. \tag{33}$$

As for the second term in Eq. (30), we notice that for similar reasons the sum on σ can be rewritten as

$$\sum_{\sigma}(1-\sigma_i)\,q(\sigma)\,\delta_{\sigma_i\sigma_j} = \sum_{\sigma} q(\sigma)\,\delta_{\sigma_i\sigma_j} - \sigma_i\,q(\sigma)\,\delta_{\sigma_i\sigma_j} = X_{ij} - Y_{ij}\ , \tag{34}$$

$$X_{ij} := \sum_{\sigma} q(\sigma)\,\delta_{\sigma_i\sigma_j} = 2Q_iQ_j - Q_i - Q_j + 1\ . \tag{35}$$

Plugging these results into the log-likelihood term, we obtain:

$$\sum_{\sigma} q(\sigma)\,\log P(A|\sigma,\theta) = \sum_{ij}\left[Y_{ij}\log \mathrm{Pois}(A_{ij};S_{ij}) + (X_{ij}-Y_{ij})\log \mathrm{Pois}(A_{ij};M_{ij}) + (1-X_{ij})\log \mathrm{Pois}(A_{ij};\delta_0)\right]\ , \tag{36}$$

and the whole $\mathscr{L}$ in Eq. (29) now is

$$\mathscr{L}(q,\theta) = \sum_{ij} Y_{ij}(-S_{ij} + A_{ij}\log S_{ij}) + (X_{ij}-Y_{ij})(-M_{ij} + A_{ij}\log M_{ij}) + \sum_{ij}(1-X_{ij})(-\delta_0 + A_{ij}\log\delta_0) \tag{37}$$

$$+ \sum_i Q_i \log(\mu) + (1-Q_i)\log(1-\mu) + H(q) \tag{38}$$

$$= \sum_{ij} Q_iQ_j(-S_{ij} + A_{ij}\log S_{ij}) + \sum_{ij}(Q_iQ_j - Q_i - Q_j + 1)(-M_{ij} + A_{ij}\log M_{ij}) \tag{39}$$

$$- \sum_{ij}(2Q_iQ_j - Q_i - Q_j)(-\delta_0 + A_{ij}\log\delta_0) + \sum_i Q_i\log(\mu) + (1-Q_i)\log(1-\mu) + \sum_i H_b(Q_i)\ . \tag{40}$$

We now have all the elements for writing the EM algorithm updates. However, some additional steps are needed for defining computationally efficient equations.

In order to have write the community parameters in such a way that at the Maximization step each u_{ik} and v_{jh} is independent by all the other elements of the vectors u_i and v_j, we introduce the variational probability:

$$\rho_{ijkh} = \frac{u_{ik}v_{jh}w_{kh}}{\sum_{kh} u_{ik}v_{jh}w_{kh}}\ . \tag{41}$$

that is representing the probability of observing an interaction between the nodes i, j because of their respective belonging to communities k, h. It will be evaluated in Expectation phase. Deriving the $\mathscr{L}$ w.r.t. u_{ik} we obtain:

$$u_{ik} = \frac{\sum_{jh}(Q_iQ_j - Q_i - Q_j + 1)\,A_{ij}\rho_{ijkh}}{\sum_{jh}(Q_iQ_j - Q_i - Q_j + 1)\,v_{jh}w_{kh}}\ . \tag{42}$$

We find similar expression for v_{ih} and w_{kh}. For the remaining quantities s_i, μ, c and δ_0, maximizing the $\mathscr{L}$ leads to the updates:

$$s_i = \frac{\sum_j Q_iQ_j\,s_j\,[A_{ji} + A_{ij}] + Q_iQ_j\,[A_{ij} - A_{ji}]}{\sum_j Q_iQ_j\,[A_{ji} + A_{ij}]}\ , \quad \mu = \frac{1}{N}\sum_i Q_i\ , \tag{43}$$

$$c = \frac{\sum_{ij} Q_iQ_jA_{ij}}{\sum_{ij} Q_iQ_j e^{-\frac{\beta}{2}(s_i - s_j - 1)^2}}\ , \quad \delta_0 = \frac{\sum_{ij} A_{ij}(2Q_iQ_j - Q_i - Q_j)}{\sum_{ij}(2Q_iQ_j - Q_i - Q_j)}\ . \tag{44}$$
